# Postural control imbalance in individuals with a minor lower extremity amputation: a scoping review protocol.

**DOI:** 10.12688/f1000research.149270.1

**Published:** 2024-04-22

**Authors:** Maxime Acien, Ahmed Dami, Virginie Blanchette, Gabriel Moisan

**Affiliations:** 1 Anatomy, Universite du Quebec a Trois-Rivieres, Trois-Rivières, QC, Canada; 2 GRAN – Groupe de Recherche sur les Affections Neuromusculosquelettiques, Trois-Rivières, QC, Canada; 3 Human Kinetics, Universite du Quebec a Trois-Rivieres, Trois-Rivières, QC, Canada; 4 VITAM – Centre de recherche en santé durable, Centre intégré universitaire de santé et de services sociaux de la Capitale-Nationale, Québec, QC, Canada

**Keywords:** Ankle, Amputation, Biomechanical Phenomena, Foot, Lower extremity, Postural Balance, Review

## Abstract

**Introduction:**

Lower extremity amputations (LEA) impact the quality of life and physical abilities and increase the risk of developing secondary health complications. Current literature predominantly focuses on major LEA, leaving a gap in understanding biomechanics related to balance and postural control in minor LEA. The shifts towards increased rates of minor LEA, due to improved quality of care and patient management, highlights the need for a focused exploration of postural control deficits in this population. The scoping review will address this gap in the literature.

**Objectives:**

The purpose of the review is to synthesize current research on postural control deficits following a minor foot amputation, focusing on any resections through or distal to the ankle joint, and to evaluate interventions aimed at improving balance in affected individuals.

**Inclusion criteria:**

The review will encompass individuals of all ages who underwent a minor foot amputation, including various levels and etiologies. It will focus on quantitative data related to standing posture, ability to perform self-balanced activities of daily living, and external aids or treatments to improve postural control. The outcomes will include modifications in the sensation of balance, fall predictors, and biomechanical changes in postural control.

**Methods:**

A preliminary search of MEDLINE (PubMed) was conducted to develop a full search strategy aimed at compiling all existing scientific articles on postural control and balance in individuals with a minor LEA. A comprehensive search will be performed across multiple databases and grey literature. Two reviewers will independently extract the data. The Mixed Methods Appraisal Tool will be used to assess risk and quality.

**Discussion:**

By emphasizing the understudied aspects of postural control imbalances induced by minor LEA, the review will highlight potential areas for therapeutic intervention and contribute to a better understanding of rehabilitation for those affected.

## Introduction

Over the last decade, the annual number of lower extremity amputations (LEA) has increased considering the aging of the population and the rise of chronic diseases. ^1^ Several studies reported a decreased rate of major LEA and an increased rate of minor LEA. ^2^^,^^3^ Commonly, major LEA are characterized by an amputation proximal to the ankle joint, while minor LEA are identified by resection through or distal to the ankle joint. ^4^ The increased rate of minor LEA compared to major LEA is mainly due to team approach to manage underlying cause leading to LEA, easier access with better quality of care, timely assessment and management of ulcers, and improved community-based outreach. ^5^^–^^7^ Diabetes, vascular disease and traumatic accidents are the three main causes of LEA. ^8^ In most developed countries, the prevalence of medical conditions such as diabetes and peripheral vascular disease is constantly rising, ^9^^,^^10^ leading to a concomitant increase in the risk of LEA. ^3^^,^^10^

LEA negatively impacts the quality of life in those affected. ^11^ An important challenge for healthcare professionals, the patients, and their caregivers is to successfully restore their physical abilities and their capacity to perform activities of daily living. ^11^ Individuals who have undergone a LEA, experience alterations in their physical abilities, which may lead to an increased risk of developing secondary comorbidities such as osteoarthritis to the amputated and/or contralateral limb, ^12^ low back pain, ^13^ and even a more proximal secondary LEA. ^2^^,^^14^ Some alterations in physical abilities could result in serious long-term biomechanical and neuromuscular deficits, leading to altered postural control, ^15^ which is a direct cause of increased risks of falls. ^16^ Experiencing a fall, in an individual with a LEA significantly reduces self-confidence and perception of balance capability, increases the fear of falling, and thus impacts the quality of life. ^11^^,^^17^

Scientific literature quantifying postural control in individuals with a LEA still predominantly focuses on major LEA. ^15^^–^^18^ Balance confidence to carry out daily activities without fear of falling is known to have a significant impact on individuals with a major LEA quality of life. ^19^ Systematic reviews have also addressed topics such as balance ^20^^,^^21^ and the risk of falls ^22^ in individuals with a major LEA. However, nearly half of the LEA are minors ^2^ and there is currently a limited number of studies focusing on better understanding the biomechanical deficits associated with minor foot amputations. Previous reviews highlighted impaired walking abilities. ^23^^,^^24^ None have focused on balance and postural control and a preliminary search of PROSPERO, MEDLINE, the Cochrane Database of Systematic Reviews, and JBI Evidence Synthesis was conducted and no current or in-progress scoping reviews or systematic reviews on this topic were identified. Additionally, external aids and treatments (e.g., protheses, ankle-foot orthoses) are commonly prescribed to patients, ^25^ but current knowledge is limited regarding how these devices could mitigate the biomechanical postural impairments resulting from a minor foot amputation. Therefore, the purpose of the scoping review will be to compile published studies that have investigated postural control deficits induced by a minor foot amputation. This will map the knowledge on this topic, which is still relatively understudied in the current literature and identify gaps of knowledge in this field. It will aim to understand the changes in postural control resulting from a minor foot amputation, the external aids and treatments that have been studied with the aim of improving physical condition and quality of life after a LEA.

## Protocol

### Review questions

Considering the PCC elements (Participants, Concept, Context), this scoping review is designed to address the following research question: “ *What quantitative data are available regarding balance deficits (C) in individuals who have undergone a minor foot amputation (P), and what tools/treatments are identified as capable of modifying postural control (C)?*” The specific questions that arise from this are as follows:

- What biomechanical variables are affected by a minor foot amputation during balance tasks?
- Are there differences in an individual's ability to maintain balance on the amputated lower extremity compared to the contralateral limb?
- Are there differences in an individual's balance regarding the level of minor LEA?
- What type/nature of treatments improve postural abilities in individuals with a minor foot LEA?

These questions will guide our review to explore and synthesize the available quantitative data on balance deficits in individuals with a minor foot amputation and identify tools or treatments that may improve posture control.

### Inclusion criteria

Table 1 contains information describing the characteristics of the studies included in the review examination.

**Table 1.** Inclusion and exclusion criteria for screening selection.

	Inclusion criteria	Exclusion criteria
**Population**	Individuals of any age with a minor foot amputation, regardless of the etiology of the LEA	Non-human studies
**Type of minor foot amputation**	- Toe(s) - Ray(s) - Metatarsal-phalangeal - Transmetatarsal - Tarsometatarsal (Lisfranc) - Midtarsal (Chopart) - Ankle (Syme)	Experimental groups composed entirely of individuals with this LEA level: - Transtibial - Knee - Transfemoral - Hip
**Outcomes**	- **Balance kinetics:** GRF, pressure peak, CoP excursion in lateral and antero-posterior plans during quiet or dynamic standing - **Kinematics:** body displacement, segments movements - **Electromyography** - **Functional test** scores	Study that tested tasks unrelated to postural control
**Study design**	- Peer-reviewed original articles - Quantitative and quantitative part of mixed-method articles - Case reports/case series, dissertation/thesis, annals of congresses, conference proceedings, presentations, posters	- Qualitative studies - Study protocol - Meta-analyses, narrative, and systematic reviews
**Study availability**	Full text	

GRF = Ground Reaction Force; CoP = Center of Pression; LEA = Lower Extremity Amputation.

**Participants.** Studies involving individuals of all ages who have undergone a minor foot amputation at the ankle joint or at a more distal level, ^4^ will be included in this review. These levels of LEA will be considered for inclusion:

• Ankle disarticulation (Syme).
• Midtarsal disarticulation (Chopart joint).
• Tarsometatarsal disarticulation (Lisfranc joint).
• Transmetatarsal amputation.
• Ray amputation.
• Metatarsal-phalangeal disarticulation.
• Toe amputation (one or more toe(s)).

No exclusion criteria will be applied regarding the etiology of LEA such as diabetes, vascular diseases (e.g., arteritis, burger disease, etc.), infectious diseases, tumors, congenital conditions, mechanical trauma (e.g., road or work accidents, etc.), or thermal trauma (e.g., burns, frostbite, etc.). Moreover, no exclusion will be made based on the unilateral or bilateral nature of the LEA. If studies included individuals who had a major LEA on one side and a minor LEA on the other, they will be considered as long as the analysis of postural control was performed separately for each lower extremity. Potential studies may address both unilateral and bilateral LEA, regardless of the level of LEA, whether it occurs in the ankle joint or more distally. If a study focuses on LEA in general, it will be included in the review if one or more clearly identified subgroups meet the inclusion criteria. No restrictions will be made based on sex, gender, race, geographic or ethnic origin of the participants.

**Concept.** This review will consider studies that quantitatively investigated balance abilities and postural control in one or more individuals who have undergone a minor foot amputation. We will gather information on possible postural biomechanical deficits identified and documented in the current literature as being induced by such LEA. The variables of interest are those related to changes in balance sensation and postural control quantitatively based on experimental conditions measured in the relevant articles. Thus, we will include studies investigating:

- Changes in balance sensation, ability to maintain a balanced posture, as well as the risk of falls. Examples of assessments will include ordinal balance scales (e.g., *Tinetti Performance Oriented Mobility Assessment* (POMA), Berg Balance Scale (BBS), etc.) or more dynamic functional tests (e.g., *Functional Reach Test* (FRT), *Timed Up and Go Test* (TUG), etc.). ^26^ These tests are indicative, and additional assessments will be considered to evaluate individual's capacity to safely balance during predetermined tasks.
- Biomechanical changes measured in a research laboratory during controlled tests. These changes included spatiotemporal aspects (e.g., time and velocity), kinematics (e.g., joint angles and accelerations, body displacement and postural oscillations), kinetics (e.g., ground reaction force, plantar pressures, center of pressure excursion in the mediolateral and anteroposterior planes, distribution/projection of the center of mass), and electromyographic changes, depending on the studies.

**Context.** This review will consider studies that have explored means implemented to improve the function deteriorated by a minor LEA, from an orthopedic perspective including prosthetics, foot/ankle orthoses, orthopedic insoles, and shoes, as well as sensitization, training, or pre/post-operative exercises. The studies eligible for review will not be limited to any geographical location.

***Types of evidence sources.*** Published, peer-reviewed quantitative studies and quantitative parts of mixed methods studies will be considered for inclusion in this scoping review. In addition, case reports, case series, theses, annals of congresses, conference proceedings, or posters will be included. Qualitative studies, research protocols, meta-analyses, narrative editorials and comments, and systematic reviews will not be considered in the review.

## Methods

The scoping review will be conducted in accordance with the methodology developed by Arksey and O'Malley ^27^ and later revised by Levac and Colquhoun, ^28^ and in line with the Preferred Reporting Items for Systematic Review and Meta-Analysis Protocols for scoping reviews (PRISMA-ScR) ^29^ guidelines. The protocol is registered with Open Science Framework ( https://osf.io/fvqbc).

### Search strategy

The search strategy will aim to locate both published peer-reviewed original studies, quantitative, and mixed method studies. An initial limited search of MEDLINE (PubMed) was undertaken in December 2023 to confirm the feasibility of this review and pilot our search strategy in identifying studies relevant to our research question. The text words contained in the titles and abstracts of relevant articles, and the index terms used to describe the articles, were used to develop a full search strategy for PubMed ( Table 2), in collaboration with the Université du Québec à Trois-Rivières librarian. The search strategy, including all identified keywords and index terms, will be adapted for each included information source. In all cases, the first theme will include all terms related to or synonymous with “ *amputation*”, the second will concern the level of amputation, and the third will include outcomes referring to balance tasks and posture control indices in standing and sitting positions. Terms from these three themes will be combined. The reference lists of articles included in the review will be screened for potential additional studies. No restrictions related to the year of publication will be applied to the search strategies. However, the search will focus on human populations and texts published in French or English.

**Table 2.** Preliminary pilot search strategy on PubMed conducted in March 2024.

Search	*PubMed Query – March 6, 2024*	Results
#4	***#1** AND **#2** AND **#3***	2 736
#3	*" **Posture**" [MeSH Terms] OR " **Proprioception**" [MeSH Terms] OR " **Postural** **Balance**" [MeSH Terms] OR " **Standing Position**" [MeSH Terms] OR " **Sitting Position**" [MeSH Terms] OR balance OR posture OR "postural control" OR stand OR sit OR "sit-to-stand" OR "stand-to-sit" OR "quiet standing" OR "quiet stand" OR "voluntary stepping response" OR reach OR "dynamic balance" OR "single leg stand" OR "one leg stand" OR "single leg stance" OR "one leg stance" OR "two legged stand" OR "two legged standing" OR "two leg stance" OR "two legged stance" OR "double leg stance" OR "double legged stance" OR "double leg standing"*	1 361 146
#2	" **Lower Extremity**" [MeSH Terms] OR " **Ankle**" [MeSH Terms] OR " **Ankle Joint**" [MeSH Terms] OR " **Foot**" [MeSH Terms] OR " **Foot Joints**" [MeSH Terms] OR " **Tarsal Joints**" [MeSH Terms] OR " **Tarsal Bones**" [MeSH Terms] OR " **Talus**" [MeSH Terms] OR " **Calcaneus**" [MeSH Terms] OR " **Metatarsal Bones**" [MeSH Terms] OR " **Toes**" [MeSH Terms] OR “ **Toe Phalanges**” [MeSH Terms] " **Toe Joint**" [MeSH Terms] OR " **Hallux**" [MeSH Terms] OR minor OR "partial foot" OR "foot joint" OR foot OR ankle OR forefoot OR midfoot OR rearfoot OR hindfoot OR toe OR ray OR phalange OR hallux* OR metatars* OR intertars* OR midtars* OR transtars* OR intermetatars* OR transmetatars* OR tarsometatars* OR lisfranc OR chopart OR syme	1 612 544
#1	" **Amputees**" [MeSH Terms] OR " **Amputation**, S **urgical**" [MeSH Terms] OR " **Amputation**, **Traumatic**" [MeSH Terms] OR amputat* OR disarticulat* OR absciss* OR excis* OR resect* OR rescis*	708 312

The databases to be searched will include SPORTDiscus (EBSCO), CINAHL (EBSCO), MEDLINE (EBSCO), Cochrane Library (CENTRAL) and Physiotherapy Evidence Database (PEDro). Other databases that catalog online trials, such as ClinicalTrials.gov and EudraCT, will also be examined. Additionally, grey literature will be explored using the academic search engine BASE, ProQuest Dissertations and Theses (ProQuest) and Google Scholar.

### Source of evidence selection

Following the search, all identified records will be collated and uploaded into EndNote software version 21.2 (Clarivate Analytics, PA, USA) and duplicates will be removed. Following a pilot test, titles and abstracts will then be screened by two independent reviewers (MA and AD) for assessment against the inclusion criteria for the review. Potentially relevant papers will be retrieved in full text and will be assessed in detail against the inclusion criteria by two independent reviewers (MA and AD). Reasons for exclusion of full-text papers that do not meet the inclusion criteria will be recorded and reported in the scoping review. Any disagreements that arise between the reviewers at each stage of the selection process will be resolved through discussion or with a third reviewer (GM). The results of the search and the reason for exclusion will be reported in full in the final scoping review and presented in a PRISMA flow diagram. ^29^

### Mapping and data extraction

Data will be extracted from included publications by two independent reviewers (MA and AD) using a data extraction tool developed by the research team. Any disagreements that arise between the reviewers will be resolved through discussion or with a third reviewer (GM). Data will be imported into a table created in a Microsoft Excel version 16.79.1 (Microsoft Corporation, WA, USA) sheet ( Table 3). The extracted data will include specific details about the participants, concept, context, study methods, and key findings relevant to the review questions. The following data will be extracted:

• Study characteristics (journal, authors, year, country where the study was conducted).
• Study population (age, sample size, comorbidity/health status, type, level, and reason for amputation, inclusion criteria).
• Study objectives and aims.
• Study design (methods, intervention description, study duration, dependent variables).
• Key findings related to the scope of the review.
• Metatarsal-phalangeal disarticulation.
• Discussion/recommendations for future research/clinical implications.

**Table 3.** Draft data extraction sheet variables.

**Study characteristics**	Year
	Journal
	Authors
	Country
**Study population**	Age
	Sample size
	Sex
	Amputation level
	Reason for amputation
	Comorbidity/health status
	Inclusion/exclusion criteria
	Control participants
**Study objectives and aims**	
**Study design**	Measured variables/Outcomes
	Methods
	Intervention
	Duration
**Results/Key findings**	
**Discussion/Limitation**	
**Conclusion**	
**Perspectives**	

A draft extraction tool is provided and will be modified and revised as necessary during the process of extracting data from each included paper. Modifications will be detailed in the full scoping review. Authors of papers will be contacted to request missing or additional data, where required.

### Data analysis and presentation

The quality of the selected studies will be evaluated using the open-access Mixed Methods Appraisal Tool ( *MMAT*) developed by Hong and colleagues. ^30^ Depending on the study type, each component will be assessed as ‘ *yes*’, ‘ *no*’, or ‘ *can't tell*’ by the two reviewers (MA and AD), and comments may be added to justify the response. In both cases, we will provide a detailed presentation of the scores attributed to each criterion, as well as an overall score based on the evaluation of all criteria, following the methodological guidelines provided by the authors. ^30^ Results will thus be contrasted according to their results. However, a poor methodological quality score will not be considered in determining inclusion. This evaluation will be performed retrospectively after the studies selection.

The results of the review will be analyzed and displayed in tabular format using charting methods to meet the objective of this scoping review. The results will be presented in a narrative format.

### Ethical considerations and dissemination

Given the nature of a scoping review, which involves synthesis and analysis of existing literature, ethical approval is not required. However, it is important to ensure that all data analysed is from reputable sources and that all referenced works are properly attributed. The results of this scoping review will be disseminated through academic channels, including peer-reviewed publications and presentations at relevant conferences. This approach will ensure that the findings can contribute to the wider scientific community and encourage further research and discussion on the topic. In addition, the review will follow the PRISMA-ScR ^29^ guidelines to ensure transparency and repeatability of the methodology used.

### Study status

We have completed our search strategy and development of keywords and medical subject headings based on the different databases. Literature searching and article extraction will begin once our search strategy has been peer reviewed.

## Discussion

The postural mechanisms affected by minor foot amputations have received limited attention in the current scientific literature. Our understanding of balance deficits in individuals with a minor foot amputation during daily activities is primarily based on extrapolation from studies conducted in individuals with a major LEA. The scoping review will map the articles that have investigated balance and postural control in one or more individuals who have lost a minor part of their lower extremity. The scoping review will provide an overview of the orthoses, prostheses, technical aids, and other treatments or modalities that have been studied in people with a minor LEA to restore or improve their postural control abilities. Synthesizing knowledge in this area and reviewing of existing treatments, with a particular focus on their impact on balance stability tasks, are essential to better assist individuals who have undergone minor LEA. Therefore, it is important to emphasize the understudied aspects of postural control imbalances induced by these amputations. The review will highlight potential areas for therapeutic intervention and contribute to a better understanding of the rehabilitation for these individuals.

### Ethics and consent

Ethical approval and written consent were not required.

## Authors contributions

**Maxime Acien.** Conceptualization, Methodology, Investigation, Writing - Original Draft, Visualization.

**Ahmed Dami.** Writing - Review & Editing.

**Virginie Blanchette.** Funding acquisition, Writing - Review & Editing.

**Gabriel Moisan.** Supervision, Project administration, Funding acquisition, Writing - Review & Editing.

All authors approved the final draft.

## Data Availability

No data is associated with this article. Open Science Framework: “Postural Control Imbalance in Individuals with a Minor Foot Amputation: A Scoping Review Protocol” ( https://doi.org/10.17605/OSF.IO/FVQBC). ^31^ Data are available under the terms of the Creative Commons Attribution 4.0 International license (CC-BY 4.0). Open Science Framework: PRISMA-ScR protocol reporting items ^32^ for “Postural Control Imbalance in Individuals with a Minor Foot Amputation: A Scoping Review Protocol” ( https://doi.org/10.17605/OSF.IO/N49TH).

## Software availability

Zotero is a free, open source, cross-platform bibliographic reference management software that can be used as an alternative to EndNote.
