# Postural control imbalance in individuals with a minor lower extremity amputation: a scoping review protocol.

**DOI:** 10.12688/f1000research.149270.2

**Published:** 2025-03-04

**Authors:** Maxime Acien, Ahmed Dami, Virginie Blanchette, Gabriel Moisan

**Affiliations:** 1 GRAN – Groupe de Recherche sur les Affections Neuromusculosquelettiques, Trois-Rivières, QC, Canada; 2 Anatomy, Universite du Quebec a Trois-Rivieres, Trois-Rivières, QC, Canada; 3 VITAM – Centre de recherche en santé durable, Centre intégré universitaire de santé et de services sociaux de la Capitale-Nationale, Québec, QC, Canada; 4 Human Kinetics, Universite du Quebec a Trois-Rivieres, Trois-Rivières, QC, Canada

**Keywords:** Ankle, Amputation, Biomechanical Phenomena, Foot, Lower extremity, Postural Balance, Review

## Abstract

**Introduction:**

Lower extremity amputations (LEA) impact the quality of life and physical abilities and increase the risk of developing secondary complications. While most research focuses on major LEA, minor LEA remain understudied despite their rising incidence. These amputations alter the sensorial and mechanical properties of the foot, affecting postural control and stability. Understanding these biomechanical changes is essential for improving rehabilitation strategies.

**Objectives:**

The scoping review will synthesize current research on postural control deficits following a minor LEA, focusing on any resections through or distal to the ankle joint. It will also evaluate whether interventions, such as orthotic devices and balance rehabilitation programs, have been investigated to mitigate balance impairments in this population.

**Inclusion criteria:**

The scoping review will include studies on individuals with a minor LEA, across various age, levels, and etiologies. The scoping review will focus on quantitative data related to standing balance and postural control, dynamic functional tests, and self-reported questionnaires on balance capacity and confidence. Studies assessing interventions for postural control restoration will be analyzed separately as a secondary outcome.

**Methods:**

A preliminary search of MEDLINE (PubMed) was conducted to develop a full search strategy aimed at compiling all existing scientific articles on postural control and balance in individuals with a minor LEA. The subsequent comprehensive search will be performed across multiple databases and grey literature. Two independent reviewers will independently extract the data. The Joanna Briggs Institute Quality Assessment Tool will be used to assess risk of bias and quality of included studies.

**Discussion:**

By mapping the literature on postural control in individuals with a minor LEA, the scoping review will highlight knowledge gaps and provide guidelines for future biomechanical and postural research protocols. It will also assess the current state of therapeutic intervention research as a secondary outcome, providing insights for clinical rehabilitation strategies.

## Introduction

Over the last decade, the annual number of lower extremity amputations (LEA) has increased partly due to the aging of the population and the rising prevalence of chronic diseases such as diabetes and peripheral vascular disease. ^1^^–^^3^ Several studies have reported that the rate of major LEAs (i.e. involving amputations proximal to the ankle joint ^4^) has stabilized or even decreased, while the rate of minor LEAs (i.e. characterized by resections through or distal to the ankle joint ^4^) has steadily increased. ^5^^,^^6^ This shift can be attributed to a team approach to managing the underlying causes of LEA, easier access with better quality of care, timely assessment and management of ulcers, and improved community-based outreach. ^7^^–^^9^

A LEA significantly impacts an individual's physical function and quality of life, ^10^ challenging healthcare professionals, the patients, and their caregivers to successfully restore their physical ability and daily living functioning. ^10^ Individuals who have undergone a LEA, experience alterations in their physical abilities, which may result in serious long-term biomechanical and neuromuscular deficits. ^11^ A LEA, even if it affects the most distal part of the foot, represents structural anatomical changes that should not be underestimated. In individuals with a LEA, the loss of sensory receptors, and changes in joint and muscle structures can severely affect postural control by disrupting proprioceptive and somatosensory feedback. ^11^^–^^13^ These changes may result in disrupted postural control and weight-bearing asymmetries, which have been associated with an increased risk of developing secondary comorbidities such as osteoarthritis of the amputated and/or contralateral limb, ^14^ low back pain, ^15^ and risks of falls. ^16^ Indeed, over half of individuals with a LEA report falling at least once within the past year. ^17^ Experiencing a fall, in an individual with a LEA, in addition to the risk of physical injury, ^18^ significantly reduces self-confidence, ^19^ perception of balance capability, ^20^ and increases the fear of falling. ^17^ Consequently, these factors have an impact on quality of life and progress in the rehabilitation process. ^10^^,^^21^

Scientific literature quantifying postural control in individuals with a LEA still predominantly focuses on major LEA. ^12^^–^^22^ Balance confidence to carry out daily activities without fear of falling is known to have a significant impact on individuals with a major LEA quality of life. ^23^ Systematic reviews have also addressed topics such as balance control ^24^^,^^25^ and the risk of falls ^18^ in individuals with a major LEA. However, the incidence of minor LEAs is rising, driven by an increase in vascular complications. ^5^ Despite this trend, there remains a limited number of studies focusing on better understanding the biomechanical deficits associated with minor LEAs. Previous reviews highlighted impaired walking abilities. ^26^^,^^27^ None have focused on balance and postural control and a preliminary search of PROSPERO, MEDLINE, the Cochrane Database of Systematic Reviews, and JBI Evidence Synthesis was conducted and no current or in-progress scoping reviews or systematic reviews on this topic were identified.

Additionally, external devices and treatments (e.g., protheses, ankle-foot orthoses, etc.) are commonly prescribed in individuals with a minor LEA to address biomechanical deficits and restore functional mobility. ^28^^,^^29^ These devices have demonstrated potential to enhance postural control and reduce fall risk in populations with conditions associated with high fall risk. ^30^^,^^31^ However, the extent to which these devices could mitigate the biomechanical postural impairments caused by a minor LEA remains largely unexplored. The literature addressing their effectiveness in restoring postural stability in this population is particularly limited, highlighting a critical gap in our understanding of their role in rehabilitation. In addition to orthotic interventions, postural reeducation programs, such as balance exercises, are commonly prescribed in populations with major LEAs and have demonstrated benefits in improving stability and reducing the risk of falls. ^32^ It is therefore plausible that similar strategies could be adapted to the specific needs of individuals with minor LEAs, although this remains poorly documented in the existing literature.

Therefore, the purpose of the scoping review will be to compile published studies that have investigated postural control deficits induced by a minor LEA. This will map the existing knowledge on this topic, which is still relatively understudied in the current literature and identify knowledge gaps in this area. The scoping review will aim to understand the changes in postural control resulting from a minor LEA. As a secondary objective, this review will examine whether included studies have evaluated interventions aimed at addressing postural control impairments in this population. These interventions may involve orthotic devices as well as postural rehabilitation programs. This dual approach will not only summarize current evidence but also provide insights into potential strategies with the aim of improving physical condition and quality of life after a minor LEA.

## Protocol

### Review questions

Considering the PCC elements (Participants, Concept, Context), this scoping review is designed to address the following research question: “ *What quantitative data are available regarding balance deficits (C) in individuals who have undergone a minor LEA (P), and what tools/treatments are identified as capable of modifying postural control (C)?*” The specific questions that arise from this are as follows:

- What biomechanical variables are affected by a minor LEA during balance tasks?
- Are there differences in an individual's ability to maintain balance on the amputated lower extremity compared to the contralateral limb?
- Are there differences in an individual's balance regarding the level of minor LEA?
- What type/nature of treatments improve postural abilities in individuals with a minor foot LEA?

These questions will guide our review to explore and synthesize the available quantitative data on balance deficits in individuals with a minor LEA and identify interventions that may improve postural control.

### Inclusion criteria

**Participants.** Studies involving individuals of all ages who have undergone a minor LEA at the ankle joint or at a more distal level, ^4^ will be included in this review. These levels of LEA will be considered for inclusion:

• Ankle disarticulation (Syme).
• Midtarsal disarticulation (Chopart joint).
• Tarsometatarsal disarticulation (Lisfranc joint).
• Transmetatarsal amputation.
• Ray amputation.
• Metatarsal-phalangeal disarticulation.
• Toe amputation (one or more toe(s)).

No exclusion criteria will be applied regarding the etiology of LEA such as diabetes, vascular diseases (e.g., arteritis, burger disease, etc.), infectious diseases, tumors, congenital conditions, mechanical trauma (e.g., road or work accidents, etc.), or thermal trauma (e.g., burns, frostbite, etc.). Moreover, no exclusion will be made based on the unilateral or bilateral nature of the LEA. Potential studies may address both unilateral and bilateral LEA, regardless of the level of LEA, whether it occurs in the ankle joint or more distally. If studies included individuals who had a major LEA on one side and a minor LEA on the other, they will be considered as long as the analysis of postural control was performed separately for each lower extremity. If a study focuses on LEA in general, it will be included in the review if one or more clearly identified subgroups meet the inclusion criteria and their results are treated independently. Studies exclusively including individuals with a major LEA in their cohort (e.g., transtibial, through knee, transfemoral, hip) will not be considered. No restrictions will be made based on sex, gender, race, geographic or ethnic origin of the participants.

**Concept.** This review will consider studies that quantitatively investigated balance abilities and postural control in one or more individuals who have undergone a minor LEA. We will gather information on possible postural biomechanical deficits identified and documented in the current literature as being induced by such condition. The variables of interest are those related to changes in balance sensation and postural control quantitatively based on experimental conditions measured in the relevant articles. Thus, we will include studies investigating:

- Changes in balance sensation, ability to maintain a balanced posture, as well as the risk of falls. Examples of assessments will include ordinal balance scales (e.g., Activities-specific Balance Confidence (ABC), *Tinetti Performance Oriented Mobility Assessment* (POMA), Berg Balance Scale (BBS), etc.) ^33^^–^^36^ or more dynamic functional tests (e.g., *Functional Reach Test* (FRT), *Timed Up and Go Test* (TUG), etc.). ^34^^–^^37^ These tests are indicative, and additional assessments will be considered to evaluate individual's confidence in their own balance and capacity to safely balance during predetermined tasks.
- Biomechanical changes measured in a research laboratory during controlled tests. The variables of interest include, but are not limited to, spatiotemporal aspects (e.g., time, velocity, etc. to perform a balance task), ^38^ kinematics (e.g., joint angles, body sway, etc.), ^39^ kinetics (e.g., ground reaction force, plantar pressures, center of pressure excursion in the medial-lateral and anterior-posterior planes, center of mass displacement, etc.), ^24^^,^^40^^–^^42^ or electromyographic changes. ^43^^,^^44^ These examples are provided to illustrate the types of biomechanical data typically reported when studying balance and postural control tasks; the review will remain open to any other quantitative parameters identified in the included studies.

**Context.** Secondly, the analysis will proceed to examine whether any of the studies included in the review examined devices and procedures for rehabilitating postural function. This review will place particular emphasis on studies that have explored means implemented to improve the function deteriorated by a minor LEA, from an orthopedic perspective including prosthetics, ankle/foot orthoses, orthopedic insoles, and therapeutic shoes, as well as sensitization, training, or pre/post-operative exercises. Studies eligible for review will not be limited to any geographical location.

***Types of evidence sources.*** Published, peer-reviewed quantitative studies and quantitative parts of mixed methods studies will be considered for inclusion in this scoping review. In addition, case reports, case series, theses, annals of congresses, conference proceedings, or posters will be included. Qualitative studies, research protocols, meta-analyses, narrative editorials and comments, and systematic reviews will not be considered in the review.

## Methods

The scoping review will be conducted in accordance with the methodology developed by Arksey and O'Malley ^45^ and later revised by Levac and Colquhoun, ^46^ and in line with the Preferred Reporting Items for Systematic Review and Meta-Analysis Protocols for scoping reviews (PRISMA-ScR) ^47^ guidelines. The protocol is registered with Open Science Framework ( https://osf.io/fvqbc).

### Search strategy

The search strategy will aim to locate both published peer-reviewed original studies, quantitative, and mixed method studies. An initial limited search of MEDLINE (PubMed) was undertaken in December 2023 to confirm the feasibility of this review and pilot our search strategy in identifying studies relevant to our research question. The text words contained in the titles and abstracts of relevant articles, and the Medical Subject Headings (MeSH) terms used to describe the articles, were used to develop a full search strategy for PubMed ( Table 1), in collaboration with the Université du Québec à Trois-Rivières librarian. The search strategy, including all identified MeSH terms and keywords, will be adapted for each included information source. In all cases, the first theme will include all terms related to or synonymous with “ *amputation*”, the second will concern the level of minor LEA, ^4^ and the third will include outcomes referring to balance tasks and posture control indices in standing and sitting positions. Terms from these three themes will be combined. The reference lists of articles included in the review will be screened for potential additional studies. No restrictions related to the year of publication will be applied to the search strategies. However, the search will focus on human populations and texts published in French or English.

**Table 1.** Preliminary pilot search strategy on PubMed conducted in March 2024.

Search	*PubMed Query – March 6, 2024*	Results
#4	***#1** AND **#2** AND **#3***	2 736
#3	*" **Posture**" [MeSH Terms] OR " **Proprioception**" [MeSH Terms] OR " **Postural** **Balance**" [MeSH Terms] OR " **Standing Position**" [MeSH Terms] OR " **Sitting Position**" [MeSH Terms] OR balance OR posture OR "postural control" OR stand OR sit OR "sit-to-stand" OR "stand-to-sit" OR "quiet standing" OR "quiet stand" OR "voluntary stepping response" OR reach OR "dynamic balance" OR "single leg stand" OR "one leg stand" OR "single leg stance" OR "one leg stance" OR "two legged stand" OR "two legged standing" OR "two leg stance" OR "two legged stance" OR "double leg stance" OR "double legged stance" OR "double leg standing"*	1 361 146
#2	" **Lower Extremity**" [MeSH Terms] OR " **Ankle**" [MeSH Terms] OR " **Ankle Joint**" [MeSH Terms] OR " **Foot**" [MeSH Terms] OR " **Foot Joints**" [MeSH Terms] OR " **Tarsal Joints**" [MeSH Terms] OR " **Tarsal Bones**" [MeSH Terms] OR " **Talus**" [MeSH Terms] OR " **Calcaneus**" [MeSH Terms] OR " **Metatarsal Bones**" [MeSH Terms] OR " **Toes**" [MeSH Terms] OR “ **Toe Phalanges**” [MeSH Terms] " **Toe Joint**" [MeSH Terms] OR " **Hallux**" [MeSH Terms] OR minor OR "partial foot" OR "foot joint" OR foot OR ankle OR forefoot OR midfoot OR rearfoot OR hindfoot OR toe OR ray OR phalange OR hallux* OR metatars* OR intertars* OR midtars* OR transtars* OR intermetatars* OR transmetatars* OR tarsometatars* OR lisfranc OR chopart OR syme	1 612 544
#1	" **Amputees**" [MeSH Terms] OR " **Amputation**, **Surgical**" [MeSH Terms] OR " **Amputation**, **Traumatic**" [MeSH Terms] OR amputat* OR disarticulat* OR absciss* OR excis* OR resect* OR rescis*	708 312

The databases to be searched will include SPORTDiscus (EBSCO), CINAHL (EBSCO), MEDLINE (EBSCO), and Cochrane Library (CENTRAL). Other databases that catalog online trials, such as ClinicalTrials.gov and EudraCT, will also be examined. Additionally, grey literature will be explored using the academic search engine BASE, ProQuest Dissertations and Theses (ProQuest) and Google Scholar.

### Source of evidence selection

Subsequent to the search, all identified records will be collated and uploaded into EndNote software version 21.2 (Clarivate Analytics, PA, USA) and duplicates will be manually removed by the first reviewer (MA). Following a pilot test, titles and abstracts will then be screened by two independent reviewers (MA and AD) for assessment against the inclusion criteria for the review. Potentially relevant papers will be retrieved in full text and will be assessed in detail against the inclusion criteria by two independent reviewers (MA and AD). Reasons for exclusion of full-text papers that do not meet the inclusion criteria will be recorded and reported in the scoping review. Any disagreements that arise between the reviewers at each stage of the selection process will be resolved through discussion or with a third reviewer (GM). The results of the search and the reason for exclusion will be reported in full in the final scoping review and presented in a PRISMA flow diagram. ^47^

### Mapping and data extraction

Data will be extracted from included publications by two independent reviewers (MA and AD) using a data extraction tool developed by the research team. Any disagreements that arise between the reviewers will be resolved through discussion or with a third reviewer (GM). Data will be imported into a table created in a Microsoft Excel version 16.79.1 (Microsoft Corporation, WA, USA) sheet ( Table 2). The extracted data will include specific details about the participants, concept, context, study methods, and key findings relevant to the review questions. The following data will be extracted:

• Study characteristics (journal, authors, year, country where the study was conducted).
• Study population (age, sample size, comorbidity/health status, type, level, and reason for amputation, inclusion criteria).
• Study objectives and aims.
• Study design (methods, intervention description, study duration, dependent variables).
• Key findings related to the scope of the review.
• Discussion/recommendations for future research/clinical implications.

**Table 2.** Draft data extraction sheet variables.

**Study characteristics**	Year
	Journal
	Authors
	Country
**Study population**	Age
	Sample size
	Sex
	Amputation level
	Reason for amputation
	Comorbidity/health status
	Inclusion/exclusion criteria
	Control participants
**Study objectives and aims**	
**Study design**	Measured variables/Outcomes
	Methods
	Intervention
	Duration
**Results/Key findings**	
**Discussion/Limitation**	
**Conclusion**	
**Perspectives**	

A draft extraction tool is provided and will be modified and revised as necessary during the process of extracting data from each included paper. Modifications will be detailed in the full scoping review. The corresponding authors of the studies will be contacted to request missing or apparently erroneous data, where required and to obtain further information on a potentially misunderstood section. If the corresponding author does not respond, the article will still be included, but problematic results will not be analyzed, and limitations will be mentioned in the discussion.

### Data analysis and presentation

The quality of the selected studies will be evaluated using the open-access Joanna Briggs Institute (JBI) Critical Appraisal Tools. The JBI Critical Appraisal Tool is designed to systematically assess the risk of bias in studies included in scoping and systematic reviews. These checklists are tailored to specific study designs (e.g., randomized controlled trials, cohort studies, quasi-experimental studies, etc.) to assess the limitations and risks of methodological bias. The tools ensure a rigorous, transparent, and standardized assessment process that focuses on internal validity by identifying potential sources of bias across domains such as participant recruitment, intervention administration, and outcome measurement. ^48^^,^^49^ Depending on the study design, each component will be assessed as ‘ *yes*’ ( *1*), ‘ *no*’, ‘unclear’, or ‘ *n/a*’ ( *0*) by the two reviewers (MA and AD), and comments may be added to justify the response. We will provide a detailed presentation of the assessments for each evaluation criterion, along with an overall rating based on the assessment of all criteria, in accordance with the methodological guidelines outlined in the JBI Framework. Results will thus be contrasted according to their results. However, a poor methodological quality score will not be considered in determining inclusion. This evaluation will be performed retrospectively after the studies selection.

The results of the studies included in the scoping review will be analyzed and presented in tabular format using charting methods to meet the objective of this scoping review. The findings will be presented in a narrative format.

### Ethical considerations and dissemination

Given the nature of a scoping review, which involves synthesis and analysis of existing literature, ethical approval is not required. However, it is important to ensure that all data analyzed is from reputable sources and that all referenced works are properly attributed. The results of this scoping review will be disseminated through academic channels, including peer-reviewed publications and presentations at relevant conferences. This approach will ensure that the findings can contribute to the wider scientific community and encourage further research and discussion on the topic. In addition, the review will follow the PRISMA-ScR ^47^ guidelines to ensure transparency and repeatability of the methodology used.

### Study status

We have completed our search strategy and development of keywords and MeSH terms based on the different databases. Literature searching and article extraction will begin once our search strategy has been peer reviewed.

## Discussion

The postural mechanisms affected by minor LEA have received limited attention in the current scientific literature. Our understanding of balance deficits in individuals with a minor LEA during daily activities is primarily based on extrapolation from studies conducted in individuals with a major LEA. The scoping review will map the articles that have investigated balance and postural control in one or more individuals who have lost a minor part of their lower extremity. The scoping review will provide an overview of the orthoses, prostheses, technical aids, and other treatments or modalities that have been studied in individuals with a minor LEA to restore or improve their postural control abilities. Synthesizing knowledge in this area and reviewing of existing treatments, with a particular focus on their impact on balance stability tasks, are essential to better assist individuals who have undergone a minor LEA. Therefore, it is important to emphasize the understudied aspects of postural control imbalances induced by these amputations. The review will highlight potential areas for therapeutic intervention and contribute to a better understanding of the rehabilitation for these individuals.

### Ethics and consent

Ethical approval and written consent were not required.

## Authors contributions

**Maxime Acien.** Conceptualization, Methodology, Investigation, Writing - Original Draft, Visualization.

**Ahmed Dami.** Writing - Review & Editing.

**Virginie Blanchette.** Funding acquisition, Writing - Review & Editing.

**Gabriel Moisan.** Supervision, Project administration, Funding acquisition, Writing - Review & Editing.

All authors approved the final draft.

## Data Availability

No data is associated with this article. Open Science Framework: “Postural Control Imbalance in Individuals with a Minor Foot Amputation: A Scoping Review Protocol” ( https://doi.org/10.17605/OSF.IO/FVQBC). ^50^ Data are available under the terms of the Creative Commons Attribution 4.0 International license (CC-BY 4.0). Open Science Framework: PRISMA-ScR protocol reporting items ^51^ for “Postural Control Imbalance in Individuals with a Minor Foot Amputation: A Scoping Review Protocol” ( https://doi.org/10.17605/OSF.IO/N49TH).

## Software availability

Rayyan (https://www.rayyan.ai/) is a free, online, open access, bibliographic reference management system that can be used as an alternative to EndNote.
