# Supplementary material for: Postural control imbalance in individuals with a minor lower extremity amputation: a scoping review protocol
Source: F1000Res. 2025 Mar 4;13:309. Originally published 2024 Apr 22. [Version 2] doi: 10.12688/f1000research.149270.2 (PMC12035671; doi:10.12688/f1000research.149270.2)
Supplement: Peer review history

# Reviewer report: Reviewer response for version 2

**DOI:** 10.5256/f1000research.178284.r373830

**Published:** 2025-04-25

**Authors:** Kenton R Kaufman

**Affiliations:** 1 Mayo Clinic, Rochester, USA

I commend the authors for developing an excellent protocol for a scoping review on the effects of minor lower extremity amputations on balance impairments (primary focus) and interventions for postural control restoration (secondary focus). The need for this review has clearly been identified. The proposed methods have been refined through a detailed response to previous reviewer comments.

 Additional considerations should be given to the outcome measures being collected. The authors plan to quantify balance impairments by collecting outcome data in two categories: patient-reported or performance-based.  Not all the outcome measures listed in the protocol have been validated in people with lower limb amputations (LLA).  Individuals with LLA have differing needs and capabilities. It is recommended that the authors consult a systematic review which identified outcome measures that have psychometrically validated for LLA populations (1). The authors can then report their findings in terms of outcome measures validated for the LLA population and those outcome measures that are used in other patient populations. Further, outcomes measures that demonstrate health related quality of life improvements should also be added to this scoping review.

Is the study design appropriate for the research question?

Yes

Is the rationale for, and objectives of, the study clearly described?

Yes

Are sufficient details of the methods provided to allow replication by others?

Yes

Are the datasets clearly presented in a useable and accessible format?

Yes

Reviewer Expertise:

.

I confirm that I have read this submission and believe that I have an appropriate level of expertise to confirm that it is of an acceptable scientific standard.

## References

[1] : Recommendations from the ISPO lower-limb COMPASS: Patient-reported and performance-based outcome measures. *Prosthet Orthot Int* .2023;47(1) : 10.1097/PXR.0000000000000197 13-25 10.1097/PXR.0000000000000197 36629556 PMC9945573

# Reviewer report: Reviewer response for version 2

**DOI:** 10.5256/f1000research.178284.r369460

**Published:** 2025-04-02

**Authors:** Humberto Omana

**Affiliations:** 1 University of Western Ontario, London, Canada

I would like to thank you for allowing me the opportunity to review this revised manuscript. I only have one comment for the authors who have done great job at making revisions. Regarding the assessment of confidence, please consider that confidence is but one way in which self-efficacy can be collected in research. There is outstanding work on this field that can help you either specify the reasoning for using confidence only or expand to ascertain you capture related concepts.

 Refer to: Bandura A. Self-efficacy: Toward a unifying theory of behavioral change. Psychol Rev. 1977. doi:10.1007/978-3-319-75361-4; Bandura A. Social foundations of thought and action : a social cognitive theory / Albert Bandura. New Jersey Prentice-Hall, 1986. 1986. Otherwise, I have no other comments. Thank you.

Is the study design appropriate for the research question?

Yes

Is the rationale for, and objectives of, the study clearly described?

Partly

Are sufficient details of the methods provided to allow replication by others?

Yes

Are the datasets clearly presented in a useable and accessible format?

Not applicable

Reviewer Expertise:

Literature reviews, Rehabilitation research, Falls prevention, Biomechanics

I confirm that I have read this submission and believe that I have an appropriate level of expertise to confirm that it is of an acceptable scientific standard.

## References

[1] : Self-efficacy: toward a unifying theory of behavioral change. *Psychol Rev* .1977;84(2) : 10.1037//0033-295x.84.2.191 191-215 10.1037//0033-295x.84.2.191 847061

# Reviewer report: Reviewer response for version 1

**DOI:** 10.5256/f1000research.163716.r306690

**Published:** 2024-09-06

**Authors:** Humberto Omana

**Affiliations:** 1 University of Western Ontario, London, Canada

Thank you for allowing me the opportunity to review this manuscript. Overall, I believe that there are important changes in both the writing and methodology that should be considered. The writing and rationale would benefit from added information. For example, knowing the biomechanics viewpoint used, then there should be writing that details why people living with LEAs have worse balance and gait. If intervention studies are also part of the scoping review, then the introduction should reflect this information. Due to the numerous ways in which balance control can be assessed, the authors would benefit from using a balance framework to present their findings. The authors need to also better explain why the MMAT is being used (for intervention studies also?). Below I will detail my feedback to the authors for consideration:

 Abstract: The argument for the need to complete this scoping review is not fully developed. What is it about a minor LEA that leaves people with a lower ability to maintain balanced? This is something that I will bring up again in the Introduction section.

 Abstract: What is the argument for including people of all ages? Can you really generalize your results to all people living with a minor LEA? This, knowing the research presented at the top of the abstract is likely related to middle aged or older adults...

 Abstract: The abstract is specific to balance control but from reading the protocol I know that interventions studies are also being included. The reasoning for this addition into the scoping review is never explained.

 Introduction: "Some alterations in physical abilities could result in serious long-term biomechanical and neuromuscular deficits, leading to altered postural control, which is a direct cause of increased risks of falls." : This sentence is vague. The authors need to explain the reason why people living with LEAs have impaired balance (how does this change according to levels of amputation?). The stance of the manuscript appears to be from neuromechanics in which canse that viewpoint should be used.

 Introduction: "Experiencing a fall, in an individual with a LEA significantly reduces self-confidence and perception of balance capability, increases the fear of falling, and thus impacts the quality of life.": Falls also lead to physical injuries and set backs regarding rehab. Although falls are mentioned throughout it would be difficult for the reader to understand how people living with LEAs would compare relative to other populations. Consider sharing falls rate. 

 Introduction: "However, nearly half of the LEA are minors...": As in younger adults? minor amputations? Revise. 

 Introduction: "Therefore, the purpose of the scoping review will be to compile published studies that have investigated postural control deficits induced by a minor foot amputation.": The introduction needs to better explain what do know about balance control in people living with LEAs, and then explain what you think you will find through your scoping review. 

 Methods: "...and what tools/treatments are identified as capable of modifying postural control (C)?”: This was never explained in the abstract or the introduction, and makes this scoping review much bigger than what was initially stated. What is the reasoning behind this? If this is one of the goals then I would expect that the gaps in the literature would also be shared.

 Methods (Table 1): The outcomes shared are broad. My suggestion would be to organize the scoping review based on a framework to help you with the many different types of variables you will come upon. Consider reviewing the following manuscripts:[1],[2],[3],[4],[5],[6].

 Methods: There is a lot of redundancy between what is on the tables and what is on the text of the methods. Please make revisions to have one or the other.

 Methods: By index terms do you mean MeSH terms?

 Methods (Table 2): This search bubble is problematic because most studies will state amputation + balance but not the level of amp. Why not combine bubbles 2 and 3 using OR? The number of items found for the combined bubbles does not seem high... but his only one database.

 Methods: Usually for reviews we also review the reference section of previous literature reviews and relevant papers. Consider adding this into your methodology as it is best practices. 

 Methods: How will duplicates be removed? If an automatic system is used, please consider also manually reviewing the removed articles as automated system can sometimes remove articles in error (which increases the more articles you gather). 

 Methods: "Authors of papers will be contacted to request missing or additional data, where required.": How? How many contact emails will be sent? What if they don't answer?

 Methods: "...Mixed Methods Appraisal Tool (MMAT)": Can you expand on this tool? what criterions does it cover? validity? for intervention studies you will be using the same tool?

Is the study design appropriate for the research question?

Yes

Is the rationale for, and objectives of, the study clearly described?

Partly

Are sufficient details of the methods provided to allow replication by others?

Yes

Are the datasets clearly presented in a useable and accessible format?

Not applicable

Reviewer Expertise:

Literature reviews, Rehabilitation research, Falls prevention, Biomechanics

I confirm that I have read this submission and believe that I have an appropriate level of expertise to state that I do not consider it to be of an acceptable scientific standard, for reasons outlined above.

## References

[1] : Center-of-Pressure Parameters Used in the Assessment of Postural Control. *Journal of Sport Rehabilitation* .2002;11(1) : 10.1123/jsr.11.1.51 51-66 10.1123/jsr.11.1.51

[2] : Normative spatiotemporal gait parameters in older adults. *Gait Posture* .2011;34(1) : 10.1016/j.gaitpost.2011.03.024 111-8 10.1016/j.gaitpost.2011.03.024 21531139 PMC3104090

[3] : Unified theory regarding A/P and M/L balance in quiet stance. *J Neurophysiol* .1996;75(6) : 10.1152/jn.1996.75.6.2334 2334-43 10.1152/jn.1996.75.6.2334 8793746

[4] : Independent domains of gait in older adults and associated motor and nonmotor attributes: validation of a factor analysis approach. *J Gerontol A Biol Sci Med Sci* .2013;68(7) : 10.1093/gerona/gls255 820-7 10.1093/gerona/gls255 23250001

[5] : Using the systems framework for postural control to analyze the components of balance evaluated in standardized balance measures: a scoping review. *Arch Phys Med Rehabil* .2015;96(1) : 10.1016/j.apmr.2014.06.021 122-132.e29 10.1016/j.apmr.2014.06.021 25073007

[6] : Postural orientation and equilibrium: what do we need to know about neural control of balance to prevent falls?. *Age Ageing* .2006;35 Suppl 2: 10.1093/ageing/afl077 ii7-ii11 10.1093/ageing/afl077 16926210

# Response

**Published:** 2025-02-13

**Authors:** Maxime Acien

**Affiliations:** Anatomie, Universite du Quebec a Trois-Rivieres, Trois-Rivières, Québec, Canada

**Dear Reviewer**,

 We would like to express our sincere gratitude for your thorough review of our manuscript and the valuable comments and suggestions you provided. Your insights have greatly contributed to improving the quality and clarity of our work, and we truly appreciate the time and effort you dedicated to evaluating our submission.

 In this letter, we address each of your comments in detail and outline the modifications made to the manuscript. We have endeavored to incorporate your suggestions carefully and hope that the revisions align with your expectations.

 Please find below a point-by-point response to your comments, along with references to the corresponding changes in the revised manuscript.

 We would like to reiterate our gratitude for your valuable contribution, which has considerably elevated the quality of this project.

 Sincerely,

 Maxime Acien, corresponding author

 Université du Québec à Trois-Rivières, Canada

 maxime.acien@uqtr.ca

**Abstract** :

**Comment #0:** **What is the argument for including people of all ages? Can you really generalize your results to all people living with a minor LEA? This, knowing the research presented at the top of the abstract is likely related to middle aged or older adults...**

     The decision to include individuals of all ages is indeed debatable. It was made following a discussion with the research team. During the preliminary search, two articles were selected that dealt with postural control in children with congenital minor LEA. Given the expected small number of results, we could not see ourselves excluding these two studies from the review. However, it is important to note that the results of studies conducted on individuals under 18 with native LEA may not be directly applicable to adults of a certain age with minor LEA resulting from vascular disorders. Nonetheless, we believe it would be worthwhile to explore these results, as they could offer valuable insights into how postural adaptations differ according to the etiology of the amputation. It is acknowledged that a child born with a "dysfunctional" limb and raised with this disorder will not have the same neuro-mechanical adaptations as a 50-year-old adult with a foot amputation due to diabetes or a 25-year-old with a minor LEA resulting from a traumatic accident or thermal trauma. We consider these different data important to address and discuss. 

**Introduction** :

**Comment #1: *"Some alterations in physical abilities could result in serious long-term biomechanical and neuromuscular deficits, leading to altered postural control, which is a direct cause of increased risks of falls." : This sentence is vague. The authors need to explain the reason why people living with LEAs have impaired balance (how does this change according to levels of amputation?). The stance of the manuscript appears to be from neuromechanics in which case that viewpoint should be used. / Introduction: "Therefore, the purpose of the scoping review will be to compile published studies that have investigated postural control deficits induced by a minor foot amputation.": The introduction needs to better explain what do know about balance control in people living with LEAs, and then explain what you think you will find through your scoping review.***

     In response to your request, the second paragraph of the introduction has been expanded. The objective is to provide a more detailed explanation from a "neuromuscular" perspective of the structural changes induced by an amputation, even a minor one. This section was further developed in the introduction of the scoping review: 

***“A LEA significantly impacts an individual's physical function and quality of life, challenging healthcare professionals, the patients, and their caregivers to restore physical ability and daily living functioning. Individuals who have undergone a LEA, experience alterations in their physical abilities, which may result in serious long-term biomechanical and neuromuscular deficits. A LEA, even if it affects the most distal part of the foot, represents structural anatomical changes that should not be underestimated. In individuals with a LEA, the loss of sensory receptors, and changes in joint and muscle structures can severely affect postural control by disrupting proprioceptive and somatosensory feedback. These changes may result in disrupted postural control and weight-bearing asymmetries, which have been associated with an increased risk of developing secondary comorbidities such as osteoarthritis of the amputated and/or contralateral limb, low back pain, and an increased risk of falls.”***

**Comment #2:** ***"Experiencing a fall, in an individual with a LEA significantly reduces self-confidence and perception of balance capability, increases the fear of falling, and thus impacts the quality of life.": Falls also lead to physical injuries and setbacks regarding rehab. Although falls are mentioned throughout it would be difficult for the reader to understand how people living with LEAs would compare relative to other populations. Consider sharing falls rate.*****

     The comment has been duly considered. Further details have been provided regarding the physical and psychological impact of a fall, as well as the rehabilitation process for patients. The fall rate of individuals living with LEA has also been added: 

***“Indeed, over half of individuals with a LEA report falling at least once within the past year. Experiencing a fall, in an individual with a LEA, in addition to the risk of physical injury, significantly reduces self-confidence, perception of balance capability, and increases fear of falling. Consequently, these factors have an impact on quality of life and progress in the rehabilitation process.”***

**Comment #3:** ***"However, nearly half of the LEA are minors...": As in younger adults? minor amputations? Revise.*****

     This segment of the sentence was not worded effectively and was challenging to generalize. While the subject has been addressed in the first paragraph, we have chosen to emphasize the increase in the incidence of minor amputations and introduce that the majority of scientific literature still focuses on major amputations. 

***“However, the incidence of minor LEAs is rising, driven by an increase in vascular complications. Despite this trend, there remains a limited number of studies focusing on better understanding the biomechanical deficits associated with minor LEAs.”***

**Methods** :

**Comment #4:** **"...and what tools/treatments are identified as capable of modifying postural control (C)?”: This was never explained in the abstract or the introduction and makes this scoping review much bigger than what was initially stated. What is the reasoning behind this? If this is one of the goals, then I would expect that the gaps in the literature would also be shared.**

     It is essential to clarify this point. The primary focus of our scoping review will be the postural control of individuals who have undergone a minor LEA. Within the scope of this "main topic," We are also exploring a secondary variable concerning the interventions implemented within this population to restore balance. While this variable was not included in the study's inclusion criteria, it will be a focal point in the review of existing interventions, including both orthopedic and physical rehabilitation approaches. This nuance was not adequately addressed in our introduction and abstract. To address this oversight, we have added several sections to the manuscript to clarify our approach.

     Firstly, an entire section has been added to the introduction:

***“Additionally, external devices and treatments (e.g., protheses, ankle-foot orthoses, etc.) are commonly prescribed in individuals with a minor LEA to address biomechanical deficits and restore functional mobility. These devices have demonstrated potential to enhance postural control and reduce fall risk in populations with conditions associated with high fall risk. However, the extent to which these devices could mitigate the biomechanical postural impairments caused by a minor LEA remains largely unexplored. The literature addressing their effectiveness in restoring postural stability in this population is particularly limited, highlighting a critical gap in our understanding of their role in rehabilitation. In addition to orthotic interventions, postural reeducation programs, such as balance exercises, are commonly prescribed in populations with major LEAs and have demonstrated benefits in improving stability and reducing the risk of falls. It is therefore plausible that similar strategies could be adapted to the specific needs of individuals with minor LEAs, although this remains poorly documented in the existing literature.”***

     In the last paragraph of the introduction on the objectives of scoping, we have added three sentences to clarify our approach and make it clear that this is a secondary objective:

***“As a secondary objective, this review will examine whether included studies have evaluated interventions aimed at addressing postural control impairments in this population. These interventions may involve orthotic devices as well as postural rehabilitation programs. This dual approach will not only summarize current evidence but also provide insights into potential strategies with the aim of improving physical condition and quality of life after a minor LEA.”***

     The "context" paragraph in the "inclusion criteria" section has also been modified to ensure clarity and avoid any potential confusion:

***“Secondly, the analysis will proceed to examine whether any of the studies included in the review examined devices and procedures for rehabilitating postural function. This review will place particular emphasis on studies that have explored means implemented to improve the function deteriorated by a minor LEA, from an orthopedic perspective including prosthetics, ankle/foot orthoses, orthopedic insoles, and therapeutic shoes, as well as sensitization, training, or pre/post-operative exercises.”***

     The new "inclusion criteria" and "discussion" paragraphs in the abstract now avoid, any confusion for the reader:

**Inclusion criteria: *“The review will include studies on individuals with a minor LEA, across various age, levels, and etiologies. The review will focus on quantitative data related to standing balance and postural control, dynamic functional tests, and self-reported questionnaires on balance capacity and confidence. Studies assessing interventions for postural control restoration will be analyzed separately as a secondary outcome.”***

**Discussion: *“By mapping the literature on postural control in individuals with a minor LEA, the scoping review will highlight knowledge gaps and provide guidelines for future biomechanical and postural research protocols. It will also assess the current state of therapeutic intervention research as a secondary outcome, providing insights for clinical rehabilitation strategies.”***

**Comment #5:** ***There is a lot of redundancy between what is on the tables and what is on the text of the methods. Please make revisions to have one or the other.*** 

     It is accurate to say that there was a significant amount of redundancy between Table 1 and the body of the text. ***Table 1 has been removed.***

**Comment #6:** ***(Table 1) The outcomes shared are broad. My suggestion would be to organize the scoping review based on a framework to help you with the many different types of variables you will come upon. Consider reviewing the following manuscripts.***

     It was challenging to compile an exhaustive list of outcomes of interest in the "concept/outcomes" section due to the numerous variables that could be studied, even if only on the Center of Pressure. The outcomes cited in this section are examples and do not constitute the complete list of our postural variables of interest, even if they were influenced by our preliminary bibliographical research.

 We have separated the outcomes into two categories: functional tests and self-reported scales, which assess balance abilities, fall risk, balance confidence, etc., and laboratory experimentation, which obtain data of a more biomechanical nature (force platforms, motion capture, etc.).

 Bibliographical references, primarily literature reviews on the variables of interest, have been included to provide readers with an objective understanding of the variables that could be of interest. These references often include studies conducted on different populations that are at high risk of falling. A sentence has been included at the end of the section to indicate the non-exhaustiveness of this list of outcomes.

***“These examples are provided to illustrate the types of biomechanical data typically reported when studying balance and postural control tasks; the review will remain open to any other quantitative parameters identified in the included studies.”***

**Comment #7:** **By index terms do you mean MeSH terms?**

     Correctly, the term "index" refers to MeSH terms, though the term "index" has been changed throughout the document.

**Comment #8:** **(Table 2) This search bubble is problematic because most studies will state amputation + balance but not the level of amp. Why not combine bubbles 2 and 3 using OR? The number of items found for the combined bubbles does not seem high... but his only one database.**

     Our search strategy is as follows: #1 AND #2 AND #3, i.e. 'the word amputation or a synonym' AND 'a level of minor amputation' AND 'a term related to balance and posture control'.

 Combining bubbles #2 and #3 with OR will result in only two bubbles, and the search strategy will be #1 AND #2, i.e., 'the word amputation or a synonym' AND 'a minor level of amputation ... OR ... a term related to balance and posture control'. At a certain point, only the terms "amputation" and "balance" will be combined, resulting in results related to posture and amputation, but not necessarily at a "minor" level (yielding numerous irrelevant results). At another point, the terms "amputation" and "amputation level" will be combined, but no terms related to postural control, resulting in many articles with the right population but the wrong outcomes.

 The number of results is notable, especially given the current state of research on postural control in individuals with a minor foot amputation.

**Comment #9:** **Usually for reviews we also review the reference section of previous literature reviews and relevant papers. Consider adding this into your methodology as it is best practices.**

     This sentence was already present in the initial manuscript in the “search strategy” section. No review currently exists on this subject. The bibliographic references of the articles included in the review will be thoroughly reviewed. No additional content has been included.

***“The reference lists of articles included in the review will be screened for potential additional studies.”***

**Comment #10:** **How will duplicates be removed? If an automatic system is used, please consider also manually reviewing the removed articles as automated system can sometimes remove articles in error (which increases the more articles you gather).**

     The articles from the various databases are loaded into the Endnote software, and duplicates are removed manually by one of the two reviewers. The sentence has been modified to clarify the situation.

***“Subsequent to the search, all identified records will be collated and uploaded into EndNote software version 21.2 (Clarivate Analytics, PA, USA) and duplicates will be manually removed by the first reviewer (MA).”***

**Comment #11:** **"Authors of papers will be contacted to request missing or additional data, where required.": How? How many contact emails will be sent? What if they don't answer?**

     In the event that any of the provided data is found to be missing or incorrect, the corresponding author will be contacted via email. Please note that failure to reply will not result in exclusion from the article. Only proven results will be discussed. A sentence has been added to clarify the section:

***“The corresponding authors of the studies will be contacted to request missing or apparently erroneous data, and to obtain further information on a potentially misunderstood section. If the corresponding author does not respond, the article will still be included, but problematic results will not be analyzed, and limitations will be mentioned in the discussion.”***

**Comment #12:** **"...Mixed Methods Appraisal Tool (MMAT)": Can you expand on this tool? what criterions does it cover? validity? for intervention studies you will be using the same tool?**

     Thank you for your feedback. The Mixed Methods Appraisal Tool was not the appropriate tool for the task at hand. We have accordingly modified the "data analysis" section. We have chosen the Joanna Briggs Institute (JBI) Critical Appraisal Tools as the new tool to be used. This will ensure a more precise fit to our specific needs and will allow for a more customized selection of assessments based on the study design.

***“The quality of the selected studies will be evaluated using the open-access Joanna Briggs Institute (JBI) Critical Appraisal Tools. The JBI Critical Appraisal Tool is designed to systematically assess the risk of bias in studies included in scoping and systematic reviews. These checklists are tailored to specific study designs (e.g., randomized controlled trials, cohort studies, quasi-experimental studies, etc.) to assess the limitations and risks of methodological bias. The tools ensure a rigorous, transparent, and standardized assessment process that focuses on internal validity by identifying potential sources of bias across domains such as participant recruitment, intervention administration, and outcome measurement. Depending on the study design, each component will be assessed as ‘yes’ (1), ‘no’, ‘unclear’ or ‘n/a’ (0) by the two reviewers (MA and AD), and comments may be added to justify the response. We will provide a detailed presentation of the assessments for each evaluation criterion, along with an overall rating based on the assessment of all criteria, in accordance with the methodological guidelines outlined in the JBI Framework.”***
